# Risks of pelvic inflammatory disease and bacterial vaginosis in adenomyosis patients using levonorgestrel intrauterine device or oral norethindrone

**DOI:** 10.3389/fendo.2025.1703310

**Published:** 2025-12-01

**Authors:** Szu-Yun Niu, Yu-Chiao Yi, Yu-Hsiang Shih, Jenn-Jhy Tseng

**Affiliations:** 1 Department of Obstetrics and Gynecology, Taichung Veterans General Hospital, Taichung, Taiwan; 2 Department of Obstetrics and Gynecology, School of Medicine, National Yang Ming Chiao Tung University, Hsinchu, Taiwan; 3 Department of Public Health, Chung Shan Medical University, Taichung, Taiwan; 4 Department of Nursing, College of Nursing, HungKuang University, Taichung, Taiwan

**Keywords:** adenomyosis, anemia, bacterial vaginosis, levonorgestrel intrauterine device, norethindrone, pelvic inflammatory disease, progesterone

## Abstract

**Background:**

Patients with adenomyosis frequently experience menorrhagia and dysmenorrhea. Levonorgestrel-releasing intrauterine device (LNG-IUD) and oral norethindrone are widely used non-surgical treatment options. However, their associated risks of pelvic inflammatory disease (PID) and bacterial vaginosis (BV) have not been well reported in the previous studies.

**Methods:**

This multi-institutional retrospective analysis was performed using de-identified electronic health records from the TriNetX Research Network. Patients with adenomyosis treated with LNG-IUD or oral norethindrone were identified. A 1:1 propensity score matching (PSM) was conducted to control for potential confounding variables. Subgroup analysis was performed to evaluate the outcomes of the patient group with hemoglobin (Hb) ≥10 g/dL or Hb <10 g/dL. Primary outcomes include risks of PID and BV. Secondary outcomes included risks of severe anemia (Hb < 10 g/dL), breast cancer, mood disorder, and cancer antigen 125 (CA-125) >35 U/mL.

**Results:**

After PSM, the LNG-IUD group showed a significantly lower risk of PID (hazard ratio (HR) 0.545; 95% confidence interval (CI) 0.483–0.616) and Hb <10 g/dL (HR 0.850; 95% CI 0.775–0.932) compared with the oral norethindrone group. In contrast, the risk of BV was significantly higher in the LNG-IUD group (HR 1.223; 95% CI 1.116–1.342). No significant differences were observed between the two groups regarding associated breast cancer, mood disorder, or CA-125 ≥ 35 U/mL.

**Conclusion:**

This study demonstrated that in patients with adenomyosis, treatment with LNG-IUD, compared with oral norethindrone, was associated with a lower risk of PID and severe anemia (Hb < 10 g/dL) but a higher risk of BV.

## Introduction

Uterine adenomyosis is characterized by the ectopic presence of endometrial glandular tissues within the myometrium and affects approximately 20% of women during their reproductive ages (1). It is associated with a variety of distressing symptoms, including cyclical pelvic pain, dyspareunia, abnormal uterine bleeding, and infertility. Conservative treatment strategies, aimed at preserving fertility and alleviating symptoms, include levonorgestrel-releasing intrauterine device (LNG-IUD), oral progestins, gonadotropin-releasing hormone analogues, combined oral contraceptives, and nonsteroidal anti-inflammatory drugs (2).

Among these options, LNG-IUD and oral progestins are commonly accepted, and their common adverse effects including abnormal menstrual bleeding, hypoestrogenic symptoms, headache, weight gain, and bone loss have been reported in previous studies, as summarized in **Supplementary Table S1** (3–8). Intrauterine device (IUD), particularly traditional copper IUD, have been linked to a higher incidence of bacterial vaginosis (BV) (9), whereas evidence regarding LNG-IUD remains inconsistent. In contrast, oral contraceptives containing progestins have been linked to a reduced risk of BV (10). With respect to pelvic inflammatory disease (PID), LNG-IUD have been shown to carry a lower risk than copper IUD (11), while oral contraceptives may also reduce PID risk, potentially due to their effects on thickening cervical mucus and decreasing menstrual flow (12, 13). However, a direct comparative evidence between LNG-IUD and oral progestins in this context is lacking.

In this study, we conducted a retrospective analysis using real-world data to compare the risks of PID and BV in adenomyosis patients treated with either LNG-IUD or oral norethindrone (primary outcomes). Secondary outcomes included risks of severe anemia (Hb < 10 g/dL), breast cancer, mood disorder, and cancer antigen 125 (CA-125) >35 U/mL.

## Methods

This retrospective, multi-institutional study utilized de-identified electronic health record data from the TriNetX Research Network (Cambridge, MA, USA), a global federated health research platform that aggregates real-time electronic health record data from healthcare organizations. Data for this study were initially extracted on April 28, 2025 from a cohort comprising approximately 126 million patients across 96 healthcare organizations primarily located in the United States and Europe. Additional subgroup analyses were performed using data extracted on November 7, 2025. Because the TriNetX database continuously updates with newly available records, the total number of patients may vary across different data extractions. The extraction dates for each dataset are provided in the footnotes of the corresponding tables. The TriNetX platform complies with the Health Insurance Portability and Accountability Act and has received a waiver from the Western Institutional Review Board due to its use of de-identified data.

**Figure 1:**
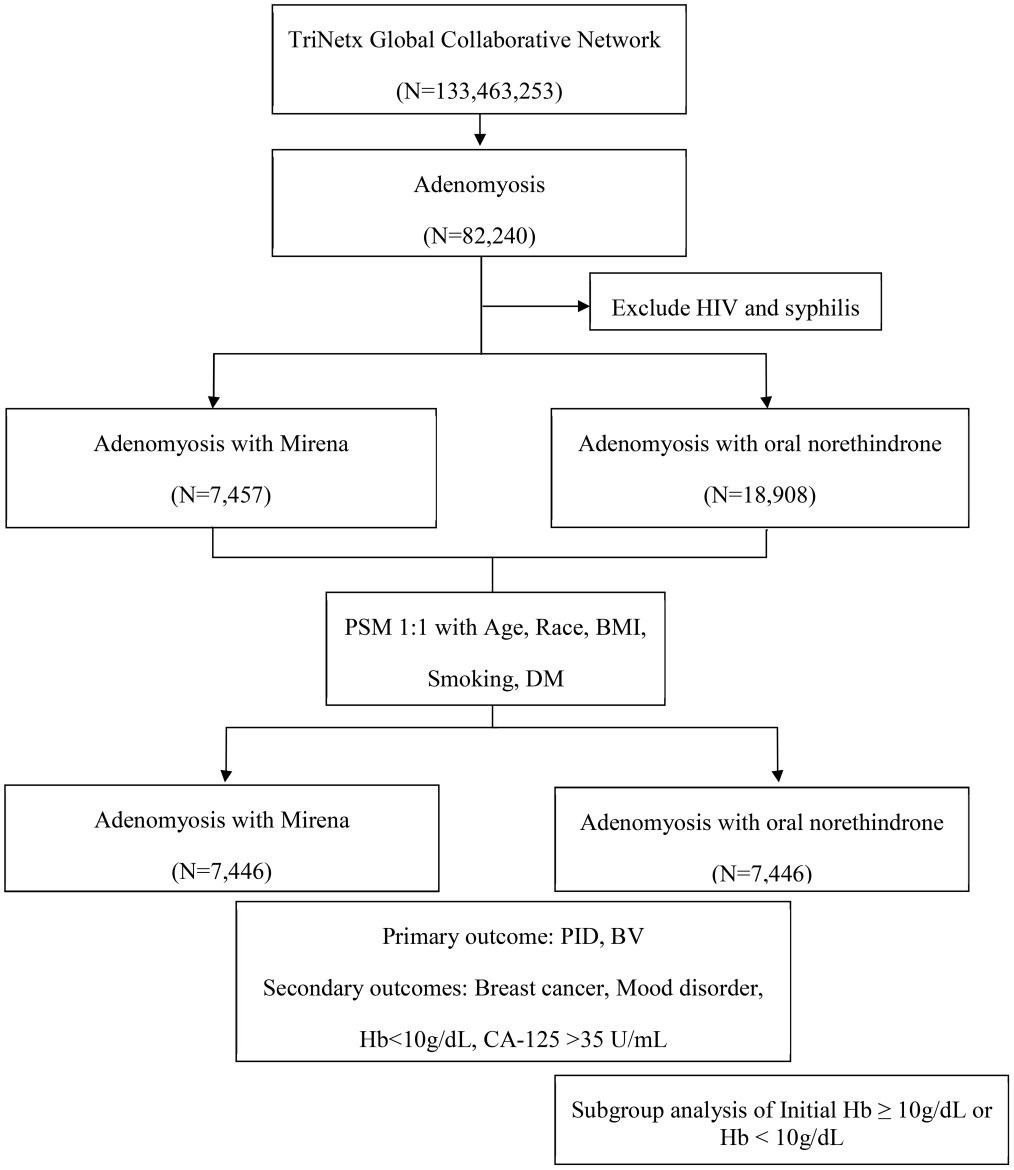
Flowchart of patient selection from the TriNetX research network. BMI, body mass index; BV, bacterial vaginosis; CA-125, cancer antigen 125; DM, diabetes mellitus; Hb, hemoglobin; HIV, human immunodeficiency virus; PID, pelvic inflammatory disease.

The study design and patient selection algorithm are illustrated in **Figure 1**. Female patients diagnosed with adenomyosis (ICD-10-CM: N80.0, N80.03) were included, excluding those with a prior history of human immunodeficiency virus (HIV) (ICD-10-CM: B20) or syphilis (ICD-10-CM: A53.9, A53). The participants were categorized into two treatment groups based on the first recorded prescription or procedure code following diagnosis: LNG-IUD insertion (Mirena, RxNorm: 6373; HCPCS: J7298) or oral norethindrone (Norina, RxNorm: 7514).

To minimize confounding factors, propensity score matching (PSM) was applied using the integrated TriNetX tool to create 1:1 matched groups with similar baseline characteristics. Patients were matched based on age, ethnicity, body mass index (BMI), smoking status (ICD-10-CM: Z72.0), and diabetes mellitus (ICD-10-CM: E08–E13, excluding E10). Ethnicity was categorized as White, Black or African American, Asian, and others or unknown. Baseline characteristics between the treatment groups were compared using chi-square test for categorical variables and *t*-test for continuous variables. A greedy nearest-neighbor matching algorithm with a caliper width of 0.1 pooled standard deviations was employed to optimize the quality of matching. A standardized mean difference (SMD) of less than 0.1 was considered indicative of a negligible difference between groups, reflecting successful matching.

The analysis period began 1 day after the initial administration of the prescribed treatment, either the LNG-IUD or oral norethindrone, and extended for 10 years. The primary outcomes of interest were the diagnoses of PID (N73, N73.1, A56.11, N73.0, A49.3, A56.19, N73.9, A74.89, N73.8, N73.2, and N97.8) and BV (B96.8 and N76.0). The secondary outcomes included mood disorder (ICD-10-CM: F32.0, F32.1, F32.2, F32.4, F32.5, F32.8, and F32.9), breast cancer (ICD-10-CM: C50, C50.91, and C50.919), Hb <10 g/dL, and CA-125 ≥35 U/mL. Following PSM, the incidence of each outcome was evaluated over a 10-year follow-up period using hazard ratio (HR) with 95% confidence interval (CI). A two-sided *p*-value of <0.05 was considered statistically significant for all analyses. Subgroup analyses were conducted to evaluate how the risk of each outcome varied according to anemia status (Hb ≥ 10 g/dL or < 10 g/dL), serving as an indirect indicator of the severity of adenomyosis.

## Results

### Baseline characteristics before and after matching

Prior to PSM, the LNG-IUD group comprised 7,457 individuals, while the oral norethindrone group included 18,908 individuals. As shown in **Table 1**, after PSM, both groups were matched 1:1, resulting in 7,446 patients in each group for subsequent analyses. The average age across both cohorts was approximately 40 years, and the mean BMI was around 30. In terms of race, the majority of patients were white, accounting for 50.3% in the LNG-IUD group and 46.5% in the norethindrone group. Regarding comorbidities, approximately 7% of patients were diagnosed with diabetes mellitus and 2.6% reported tobacco use. The matching process was considered successful as indicated by SMD below 0.1 for all matched variables, as shown in **Table 1**.

**Table 1:** Baseline characteristics of the patients enrolled.

Variables	Before matching				After matching			
	LNG-IUD (*N* = 7,457)	Norethindrone (*N* = 18,908)	*P*-value	SMD	LNG-IUD (*N* = 7,446)	Norethindrone (*N* = 7,446)	*P*-value	SMD
Age at index (mean ± SD)	39.7 ± 8.7	40.5 ± 8.5	<0.001	0.089	39.7 ± 8.7	39.5 ± 8.5	0.182	0.022
Race								
White	3,750 (50.3%)	8,795 (46.5%)	<0.001	0.076	3,750 (50.4%)	3,749 (50.3%)	0.987	<0.001
Other or unknown race	1,711 (22.9%)	3,245 (17.2%)	<0.001	0.118	1,700 (22.8%)	1,698 (22.8%)	0.964	0.001
Black or African American	1,283 (17.2%)	3,325 (17.6%)	0.465	0.010	1,283 (17.2%)	1,285 (17.3%)	0.965	0.001
Asian	713 (9.6%)	3,543 (18.7%)	<0.001	0.266	713 (9.6%)	714 (9.6%)	0.978	<0.001
BMI	30.6 ± 8.3	29.5 ± 7.7	<0.001	0.135	30.6 ± 8.3	30.2 ± 7.7	0.016	0.045
BMI < 30	3,737 (54.8%)	10,122 (59.0%)	<0.001	0.068	3,735 (54.9%)	3,814 (55.1%)	0.195	0.021
BMI ≥ 30	3,077 (45.2%)	7,047 (41.0%)	<0.001	0.082	3,072 (45.1%)	3,113 (44.9%)	0.495	0.045
Diagnoses								
Diabetes mellitus	557 (7.5%)	1,258 (6.7%)	0.018	0.032	556 (7.5%)	528 (7.1%)	0.377	0.014
Smoking	195 (2.6%)	487 (2.6%)	0.856	0.002	195 (2.6%)	172 (2.3%)	0.224	0.020

The data was extracted on April 29, 2025 (08:09, Greenwich Mean Time +8).

BMI, body mass index; BV, bacterial vaginosis; CA-125, cancer antigen 125; Hb, hemoglobin; LNG-IUD, levonorgestrel intrauterine device; PID, pelvic inflammatory disease; PSM, propensity score matching; SMD, standardized mean difference.

### Incidence of outcomes between LNG-IUD and oral norethindrone group

**Figure 2:**
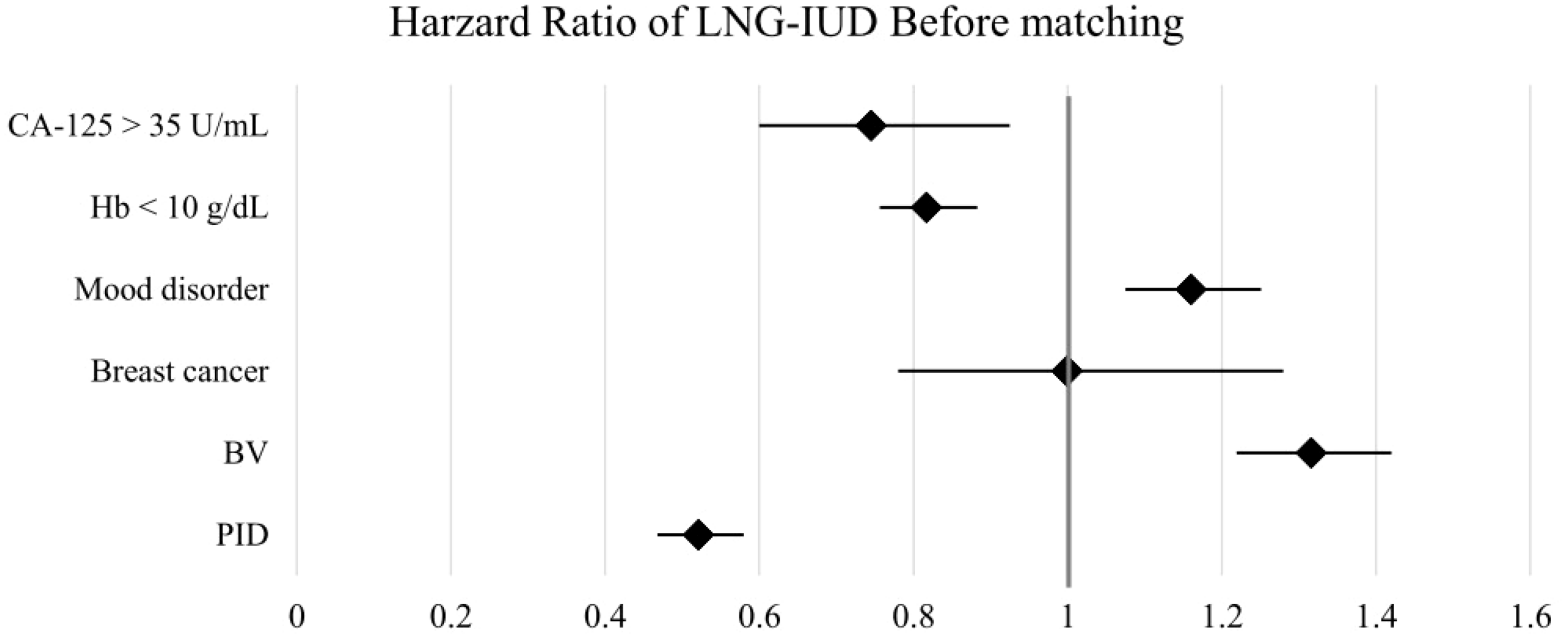
Forest plots of outcomes before matching. BV, bacterial vaginosis; CA-125, cancer antigen 125; Hb, hemoglobin; LNG-IUD, levonorgestrel intrauterine device; PID, pelvic inflammatory disease; PSM, propensity score matching.

**Figure 3:**
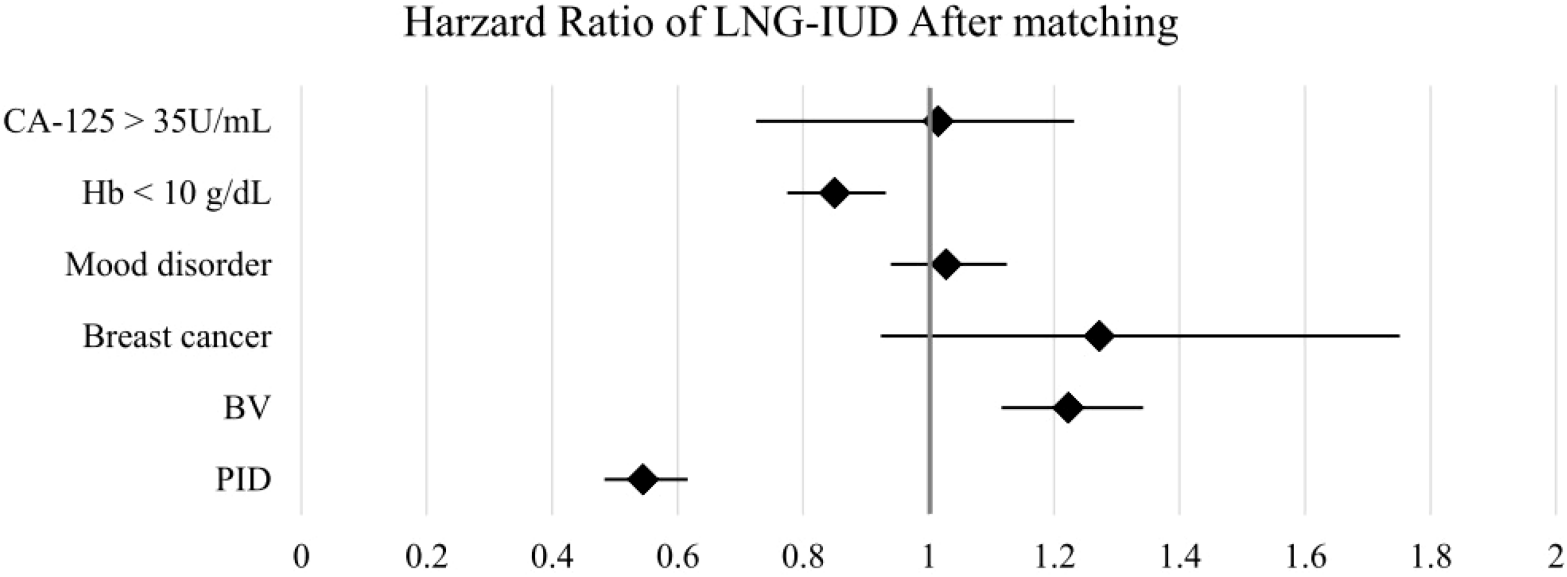
Forest plots of outcomes after matching. BV, bacterial vaginosis; CA-125, cancer antigen 125; Hb, hemoglobin; LNG-IUD, levonorgestrel intrauterine device; PID, pelvic inflammatory disease; PSM, propensity score matching.

The HR for clinical outcomes were presented in **Table 2** and **Figures 2**, **3**. Following PSM, the LNG-IUD group demonstrated a significantly lower risk of PID (HR 0.545; 95% CI 0.483–0.616) and Hb < 10 g/dL (HR 0.850; 95% CI 0.775–0.932) compared with the norethindrone group. Conversely, the risk of BV was higher in LNG-IUD group (HR 1.223; 95% CI 1.116–1.342). No statistically significant differences were observed between the two groups for other outcomes, including breast cancer, mood disorder, and CA-125 ≥35 U/mL.

**Table 2:** Outcomes before and after PSM.

Outcome	Before matching			After matching		
	LNG-IUD (*N* = 7,457)	Norethindrone (*N* = 18,908)	Hazard ratio	LNG-IUD (*N* = 7,446)	Norethindrone (*N* = 7,446)	Hazard ratio
PID	402 (5.4%)	1,906 (10.1%)	0.521 (0.468, 0.580)	401 (5.4%)	713 (9.6%)	0.545 (0.483, 0.616)
BV	991 (13.3%)	1,988 (10.5%)	1.316 (1.219, 1.420)	992 (13.3%)	828 (11.1%)	1.223 (1.116, 1.342)
Breast cancer	87 (1.2%)	222 (1.2%)	0.999 (0.780, 1.280)	86 (1.2%)	67 (0.9%)	1.272 (0.924, 1.751)
Mood disorder	965 (13.0%)	2,157 (11.4%)	1.160 (1.075, 1.251)	964 (12.9%)	938 (12.6%)	1.028 (0.940, 1.125)
Hb <10 g/dL	842 (11.3%)	2,582 (13.7%)	0.817 (0.756, 0.883)	840 (11.3%)	972 (13.1%)	0.850 (0.775, 0.932)
CA-125 >35 U/mL	106 (1.4%)	362 (1.9%)	0.745 (0.600, 0.925)	106 (1.4%)	112 (1.5%)	1.015 (0.725, 1.232)

The data was extracted on April 29, 2025 (08:09, Greenwich Mean Time +8).

BV, bacterial vaginosis; CA-125, cancer antigen 125; Hb, hemoglobin; LNG-IUD, levonorgestrel intrauterine device; PID, pelvic inflammatory disease; PSM, propensity score matching.

### Subgroup analysis by hemoglobin level

The baseline characteristics of patients stratified by Hb ≥10 g/dL or <10 g/dL are presented in **Supplementary Tables S2**, **S3**. The total number of patients in these subgroups was smaller than in the overall cohort because hemoglobin data were not available for all individuals. After PSM, the number of patients in both the LNG-IUD and oral norethindrone groups was 5,640 for the Hb ≥10 g/dL subgroup and 488 for the Hb <10 g/dL subgroup, respectively, as shown in **Table 3**. In the Hb ≥10 g/dL subgroup, the LNG-IUD group showed a significantly lower risk of PID compared with the norethindrone group (HR 0.533; 95% CI 0.465–0.611). However, the risk of BV was significantly higher in the LNG-IUD group (HR 1.119; 95% CI 1.015–1.233). No statistically significant differences were observed between the two groups for breast cancer, mood disorder, or CA-125 ≥35 U/mL. In the Hb <10 g/dL subgroup, which had a smaller sample size, the LNG-IUD group similarly exhibited a significantly reduced risk of PID (HR 0.559; 95% CI 0.341–0.917). Although BV showed a similar trend toward a higher incidence in the LNG-IUD group, the difference did not reach statistical significance (HR 1.239; 95% CI 0.819–1.874). For all other outcomes, including breast cancer, mood disorder, and elevated CA-125 levels, no statistically significant differences were observed between the treatment groups.

**Table 3:** Subgroup analysis of outcomes before and after PSM.

Hb ≥ 10 g/dL						
	Before matching			After matching		
Outcome	LNG-IUD (N = 5,643)	Norethindrone (N = 14,667)	Hazard ratio	LNG-IUD (N = 5,640)	Norethindrone (N = 5,640)	Hazard ratio
PID	321 (5.7%)	1594 (10.9%)	0.508 (0.451, 0.573)	320 (5.7%)	577 (10.2%)	0.533 (0.465, 0.611)
BV	854 (15.2%)	1791 (12.2%)	1.287 (1.186, 1.397)	855 (15.2%)	768 (13.6%)	1.119 (1.015, 1.233)
Breast cancer	70 (1.2%)	191 (1.3%)	0.958 (0.729, 1.260)	70 (1.2%)	66 (1.2%)	1.046 (0.747, 1.464)
Mood disorder	910 (16.2%)	1982 (13.5%)	1.228 (1.135, 1.328)	911 (16.2%)	867 (15.4%)	1.047 (0.954, 1.149)
Hb < 10 g/dL	578 (10.3%)	1740 (11.9%)	0.857 (0.780, 0.941)	580 (10.3%)	636 (11.3%)	0.894 (0.799, 1.001)
CA-125 > 35 U/mL	87 (1.5%)	297 (2.0%)	0.763 (0.601, 0.969)	87 (1.5%)	79 (1.4%)	1.093 (0.806, 1.482)

Hb < 10 g/dL						
	Before matching			After matching		
Outcome	LNG-IUD (N = 503)	Norethindrone (N = 1,416)	Hazard ratio	LNG-IUD (N = 488)	Norethindrone (N = 488)	Hazard ratio
PID	25 (5.0%)	123 (8.8%)	0.579 (0.377, 0.890)	24 (4.9%)	45 (9.2%)	0.559 (0.341, 0.917)
BV	49 (9.8%)	117 (8.3%)	1.262 (0.904, 1.762)	47 (9.6%)	43 (8.8%)	1.239 (0.819, 1.874)
Breast cancer	10 (2.0%)	15 (1.1%)	0.957 (0.348, 2.633)	10 (2.0%)	10 (2.0%)	0.700 (0.197, 2.482)
Mood disorder	39 (7.8%)	109 (7.8%)	1.051 (0.729, 1.514)	38 (7.8%)	39 (8.0%)	1.042 (0.667, 1.630)
Hb < 10 g/dL	288 (57.6%)	887 (63.1%)	0.894 (0.782, 1.021)	279 (57.2%)	325 (66.6%)	0.842 (0.718, 0.989)
CA-125 > 35 U/mL	18 (3.6%)	51 (3.6%)	1.042 (0.609, 1.784)	18 (3.7%)	18 (3.7%)	1.099 (0.571, 2.112)

The data was extracted on April 29, 2025 (08:09, Greenwich Mean Time +8).

BMI, body mass index; BV, bacterial vaginosis; CA-125, cancer antigen 125; Hb, hemoglobin; LNG-IUD, levonorgestrel intrauterine device; PID, pelvic inflammatory disease; PSM, propensity score matching.

## Discussion

Most existing studies comparing LNG-IUD and oral progestins have been conducted in the context of contraception (9, 10, 14). More recently, a meta-analysis evaluated the efficacy and adverse effects of LNG-IUD for adenomyosis, both as monotherapy and in combination with other treatments (15). Our study demonstrated a novel real-world study directly comparing LNG-IUD and oral norethindrone in patients with adenomyosis. Unlike contraception, the management of adenomyosis involves long-term treatment, often from a woman’s 20s through her 50s, and requires careful clinical counseling regarding both efficacy and safety (1, 2). Among these concerns, the potential risk of breast cancer is frequently raised by patients. The LNG-IUD provides a convenient, locally acting therapy; however, some patients report abdominal or vaginal discomfort following insertion. This raises important clinical questions about whether the risks of PID, BV, breast cancer, mood disorder, and disease control differ between LNG-IUD and oral norethindrone use. In our study, LNG-IUD use was associated with a reduced risk of PID and severe anemia but an increased risk of BV compared with oral norethindrone after PSM.

We observed a reduced risk of PID and severe anemia (Hb < 10 g/dL) among adenomyosis patients treated with LNG-IUD compared with those receiving oral norethindrone after PSM. The association between LNG-IUD use and PID remains controversial, with existing research limited by methodological constraints (14). While copper IUDs have been linked to an increased risk of PID (16), LNG-IUD appears to confer a lower risk compared with copper IUDs (11); however, no prior studies have directly compared LNG-IUD with oral norethindrone in this context. We hypothesize that levonorgestrel released by the LNG-IUD may exert a protective effect due to its anti-inflammatory and endometrial-thinning properties. On the other hand, clinical trials have further demonstrated that LNG-IUD significantly improves Hb levels in patients with adenomyosis, with one randomized trial showing comparable hematologic outcomes to hysterectomy but superior psychological and social well-being (17). Another randomized study found LNG-IUD to be more effective than combined oral contraceptives in reducing menstrual blood loss (18). Our findings are consistent with these results, confirming a greater improvement in anemia among adenomyosis patients treated with LNG-IUD.

Previous research has shown that norethindrone does not significantly alter the mucosal microbial environment (19), and further studies have reported a negative association between BV and the use of oral contraceptives (20, 21). In contrast, BV has been frequently linked to copper IUD use (9, 22), whereas the evidence regarding LNG-IUD remains limited and inconsistent (19). Short-term LNG-IUD use has been reported to transiently decrease lactobacillary dominance and increase BV, with risks returning to baseline after 1–5 years (23). Comparative analyses further suggest that copper IUDs are associated with higher rates of BV and *Actinomyces* species, while LNG-IUD are more often linked to candidiasis (24). Although most available data are derived from contraceptive populations, our findings similarly indicate an elevated risk of BV with LNG-IUD use compared with oral norethindrone in adenomyosis patients.

The associations of breast cancer and mood disorder remain major concerns among users of hormonal therapy. Although LNG-IUD is primarily locally acting, prior studies have reported that both oral combined estrogen–progestin contraceptives and LNG-IUD are associated with an increased risk of breast cancer compared with non-users, with risk rising in proportion to duration of use (25). A meta-analysis further suggested that LNG-IUD use may increase breast cancer risk irrespective of age or indication, with a stronger effect observed in older women (26). In contrast, our 10-year follow-up analysis found no significant difference in breast cancer incidence between LNG-IUD and oral norethindrone users.

Regarding mood disorder, the available evidence remains inconsistent. While most studies have not demonstrated a consistent adverse impact of hormonal contraceptives on mood in the general population, certain individuals may be particularly susceptible to mood-related side effects (27). LNG-IUD, in particular, have been linked to an elevated risk of depression in a UK cohort study (28), and it has been suggested that patients should be counseled regarding the potential risk of psychiatric symptoms prior to use (29). In our study, however, no significant difference in the incidence of mood disorder was observed between LNG-IUD and oral norethindrone users.

This study provided clinically relevant information to guide the counseling and management of patients with adenomyosis. For individuals with a history of recurrent BV, oral norethindrone may be the preferred treatment option. Conversely, in patients with a prior history of PID or those experiencing severe menorrhagia, LNG-IUD may be more appropriate. Regarding concerns about the risk of breast cancer and mood disorder, our findings indicated no significant differences between the two treatment modalities; however, according to previous studies, appropriate counseling of the breast cancer and psychiatric risks is essential before prescribing these medications.

In addition to these considerations, patient-specific factors should also guide treatment selection. Previous studies have reported a higher risk of LNG-IUD expulsion in obese patients (30), suggesting that norethindrone may be more suitable for individuals with higher BMI. Smoking has been associated with an increased risk of bacterial vaginosis (31), further supporting the use of oral norethindrone in smokers. Conversely, for younger women who also require contraception, the LNG-IUD may be preferred for its dual therapeutic and contraceptive benefits.

Our study has several limitations that should be acknowledged, most of which are inherent to the use of the TriNetX database. Because imaging data are not included in TriNetX, parameters such as uterine size and myometrial thickness, which may influence treatment efficacy and IUD expulsion rates, could not be evaluated. As a retrospective analysis, this study cannot establish causal relationships between treatments and outcomes, and residual confounding may remain despite propensity score matching. Detailed information on medication dosage, adherence, and treatment duration was also unavailable, which may have affected the accuracy of outcome assessment. Given that the approved duration of LNG-IUD use is 5 years, we additionally performed a 5-year follow-up analysis, which demonstrated trends consistent with those observed in the original cohort. The results are presented in **Supplementary Tables S4**, **S5**. In addition, while menorrhagia is a predominant symptom of adenomyosis, data on LNG-IUD removal, expulsion, or discontinuation were unavailable, potentially reducing the accuracy of outcome assessment. Furthermore, reliance on standardized coding systems (ICD and RxNorm) may lead to misclassification or reporting delays, particularly for conditions such as BV or mood disorder that may be underdiagnosed in clinical practice.

Future prospective studies with standardized, protocol-based follow-up assessments are warranted to provide more precise and clinically informative results. In addition, we conducted an extended exploratory analysis including patients diagnosed with both adenomyosis and concomitant endometriosis to further elucidate treatment outcomes in this subgroup. The preliminary findings, presented in **Supplementary Tables S6**, **S7**, demonstrated trends consistent with those observed in the original cohort.

Despite these limitations, this study provides an advantageous real-world evidence comparing two widely used conservative treatments for adenomyosis. The large sample size, diverse patient population, and rigorous application of PSM strengthen the validity of our findings and support their relevance for clinical decision-making.

## Conclusion

In patients with adenomyosis, treatment with LNG-IUD, compared with oral norethindrone, was associated with a reduced risk of PID and severe anemia (Hb < 10 g/dL) but an increased risk of BV. No significant differences were observed between the two treatments regarding breast cancer, mood disorder, or CA-125 >35 U/mL.

## Data Availability

The original contributions presented in the study are included in the article/**Supplementary Material**. Further inquiries can be directed to the corresponding author.
