# Supplementary figures and images for: Risks of pelvic inflammatory disease and bacterial vaginosis in adenomyosis patients using levonorgestrel intrauterine device or oral norethindrone
Source: Front Endocrinol (Lausanne). 2025 Dec 1;16:1703310. doi: 10.3389/fendo.2025.1703310 (PMC12702703; doi:10.3389/fendo.2025.1703310)

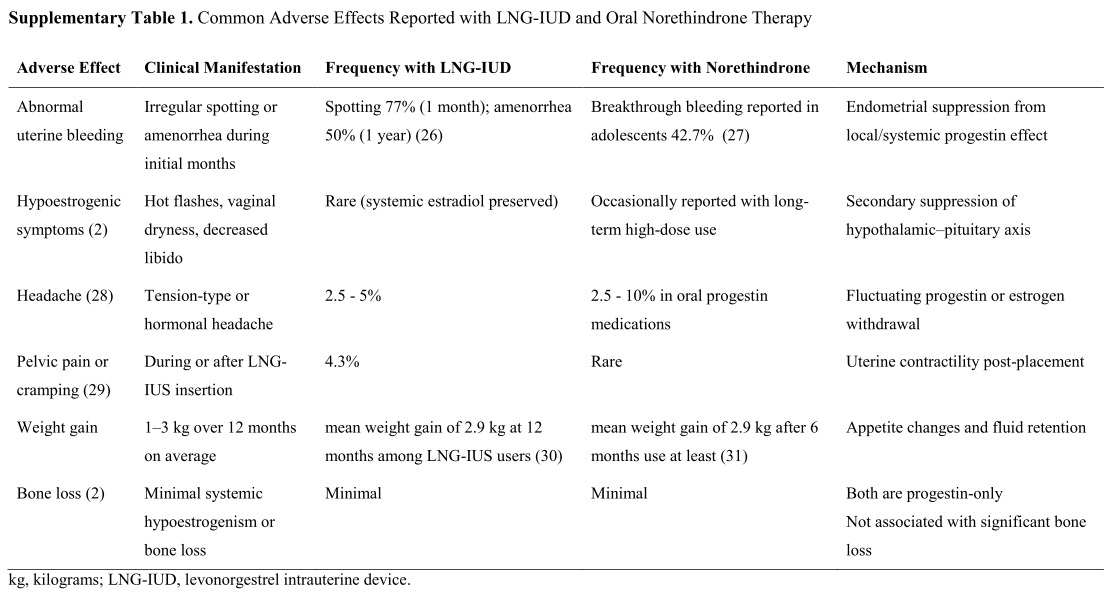

Supplement: Supplementary file 1 [file Image1.jpeg]

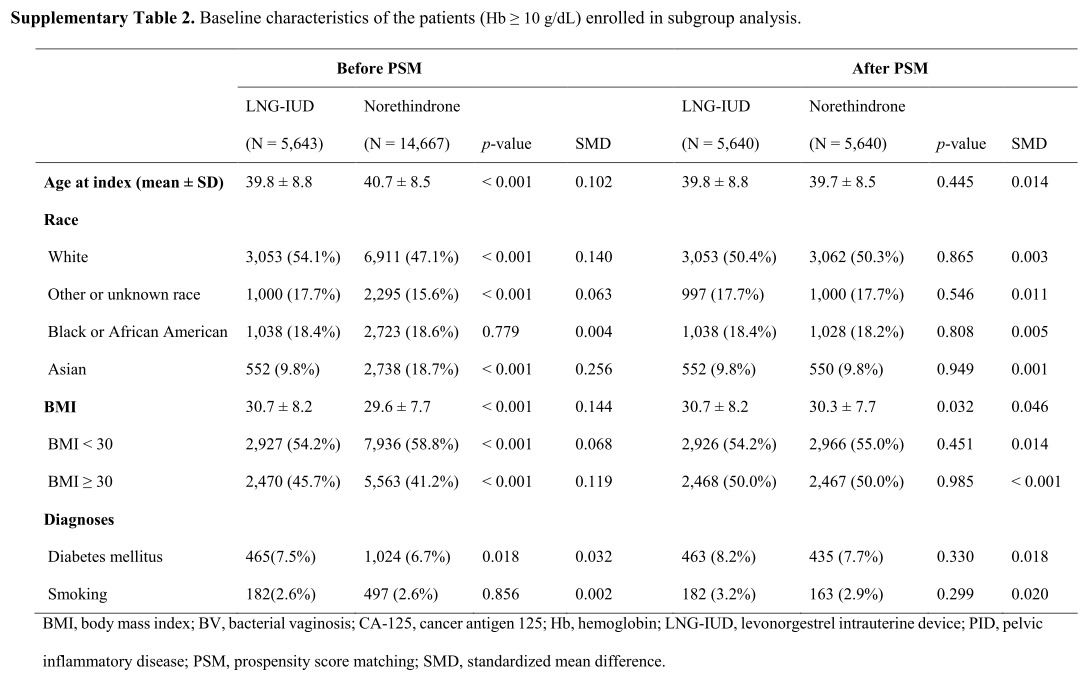

Supplement: Supplementary file 2 [file Image2.jpeg]

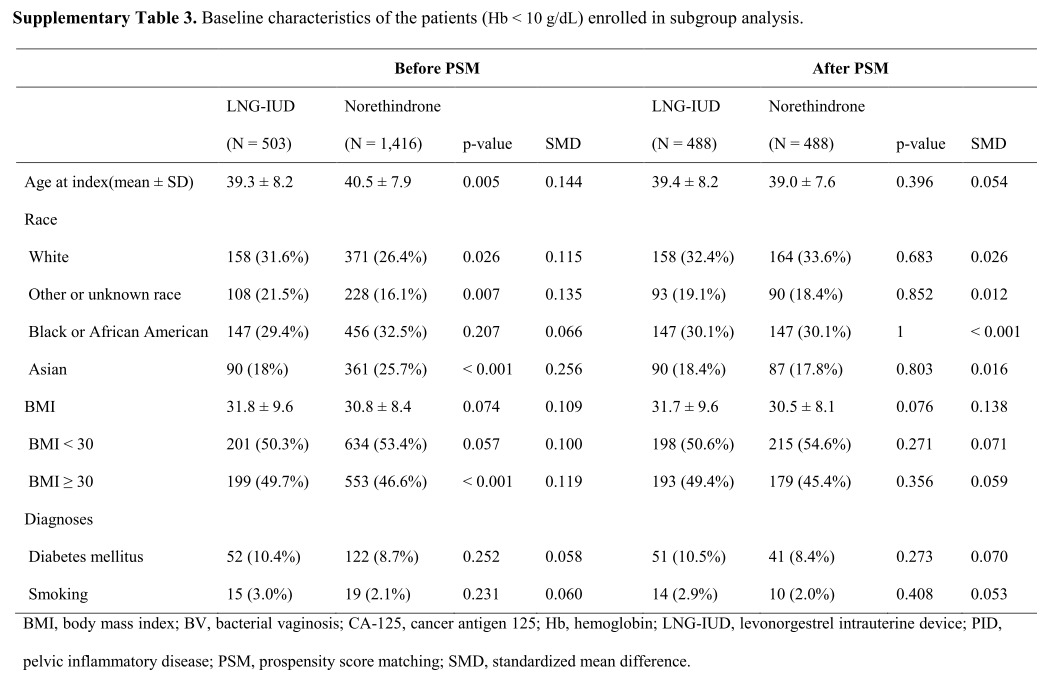

Supplement: Supplementary file 3 [file Image3.jpeg]

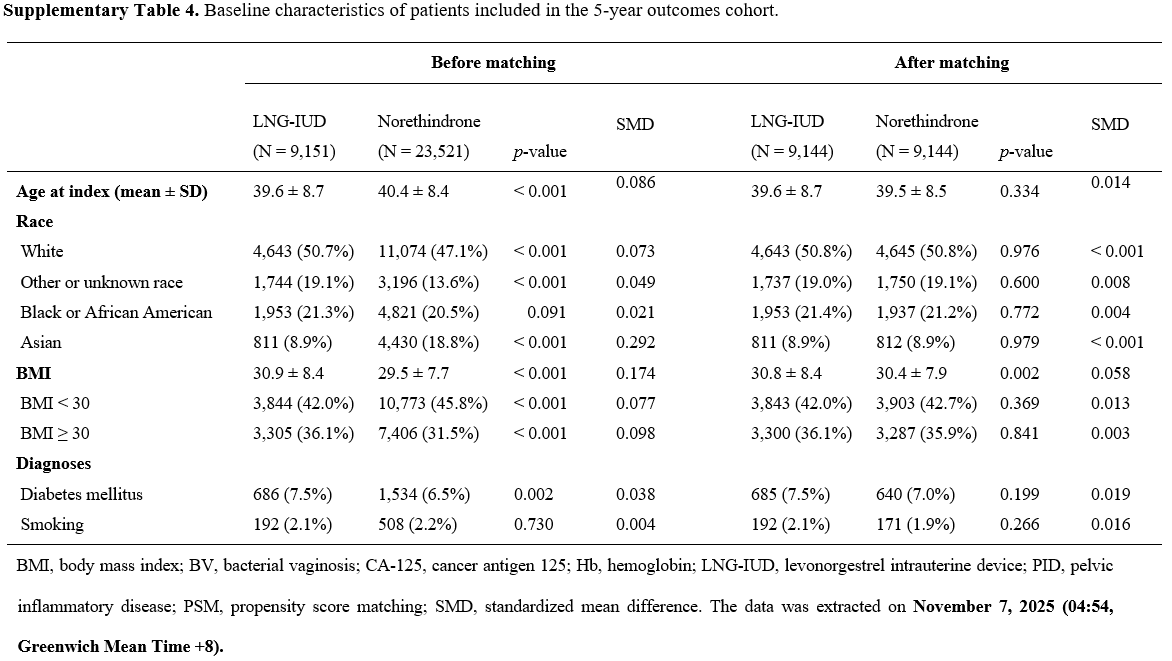

Supplement: Supplementary file 4 [file Image4.png]

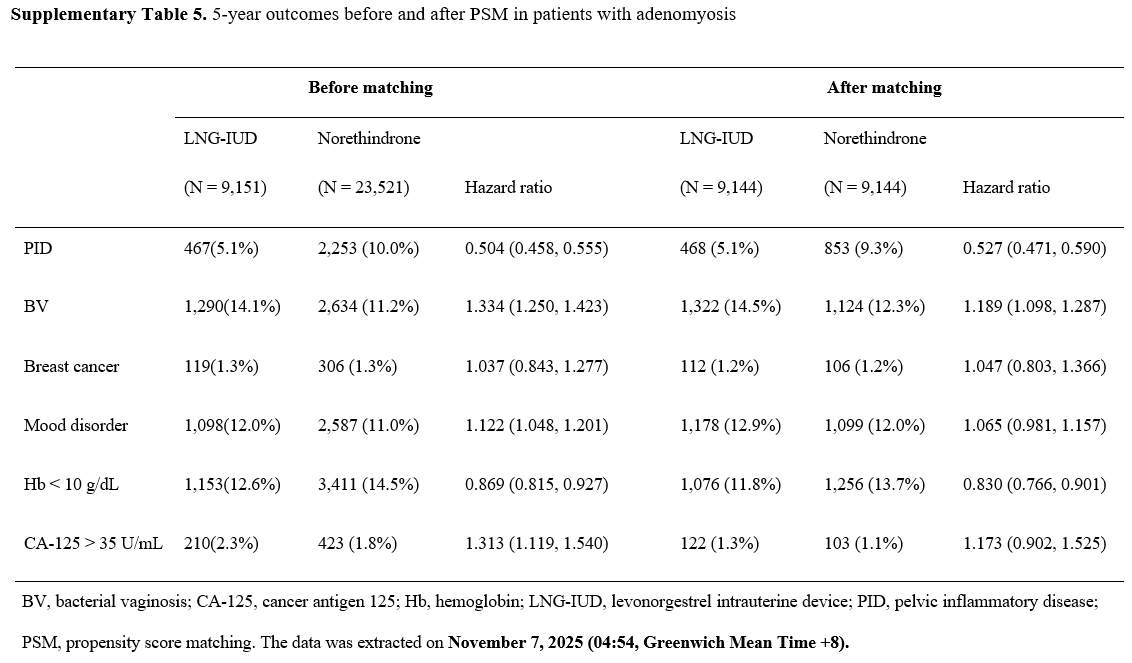

Supplement: Supplementary file 5 [file Image5.png]

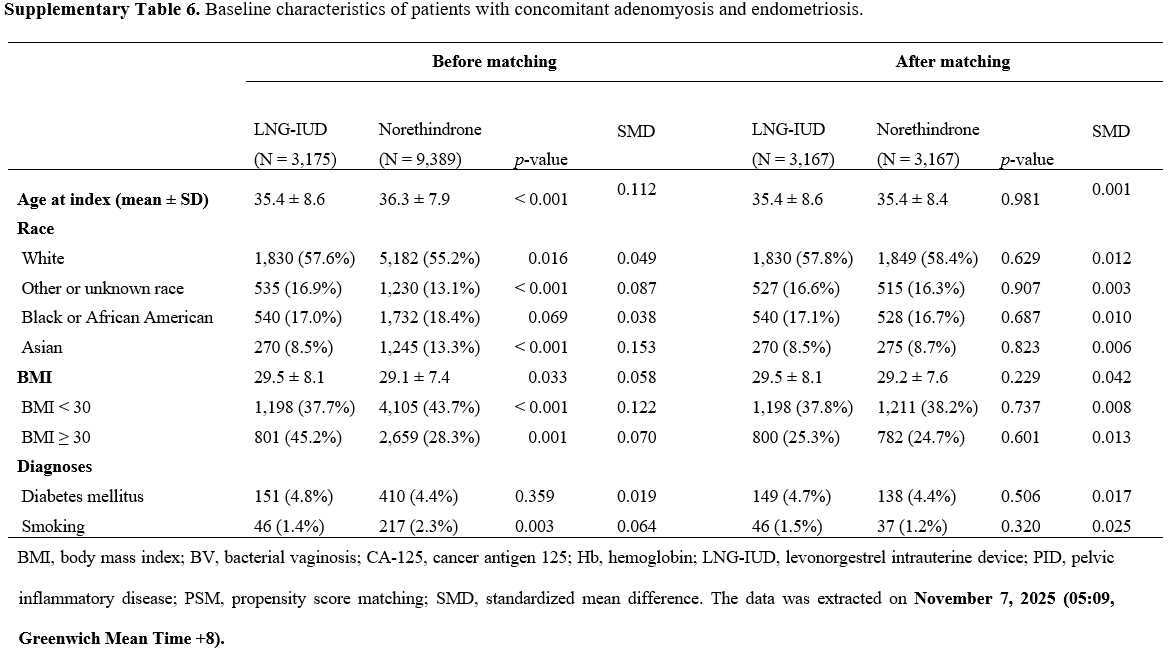

Supplement: Supplementary file 6 [file Image6.png]

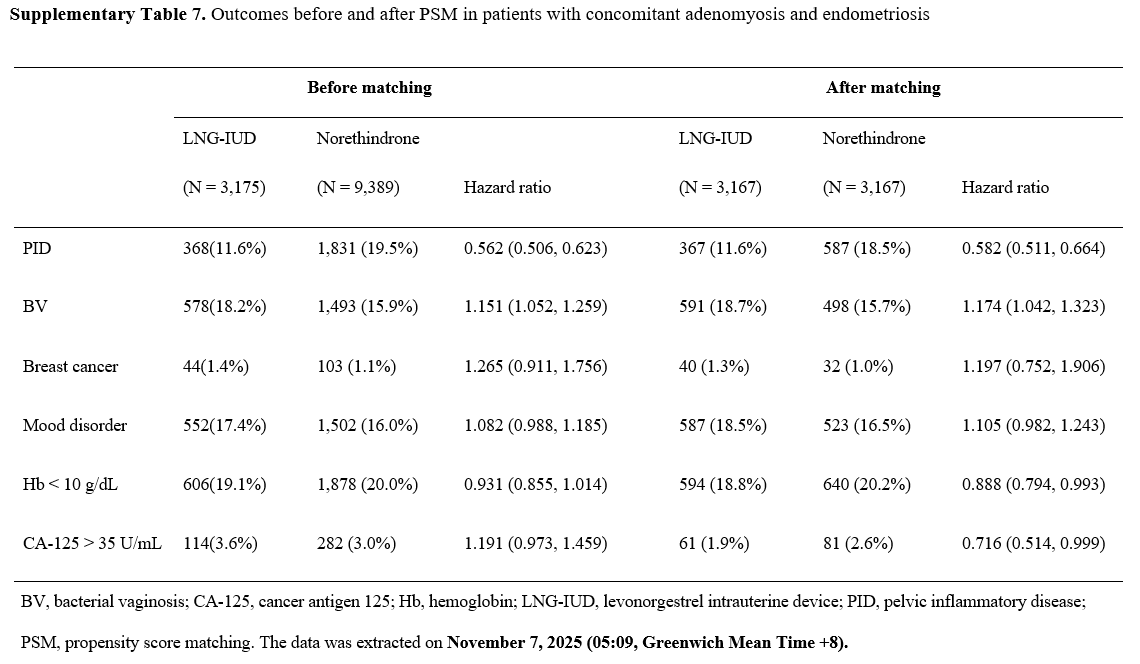

Supplement: Supplementary file 7 [file Image7.png]
